# Supplementary material for: Splice-Junction-Based Mapping of Alternative Isoforms in the Human Proteome
Source: Cell Rep. Author manuscript; Available in PMC 2020 Jan 15. (PMC6961840; doi:10.1016/j.celrep.2019.11.026)

A

sp|P27816|MAP4_HUMAN|ENSG00000047849|MXE1|1455|chr3|47914939|47917174|−2|r878|T1
MYHDDDLADLVFPSSATADTSIFAGQNDPLKDSYVPLELAK q value: 4.4154e−05 Tr_novel:TRUE RefSeq_Novel:FAL
Search result spec prec mz: 1118.5439 Actual spec prec mz: 1118.5439
Fragments matched per AA: 0.854 Proportion of top 20 peaks matched: 0.3

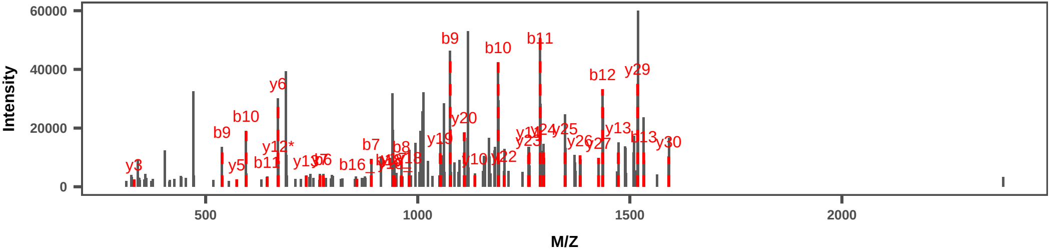

B

Scatterplot of predicted elution time
Fitting R2: 0.833
Novel peptide residual Z score: 0.185
Number of peptides: 725

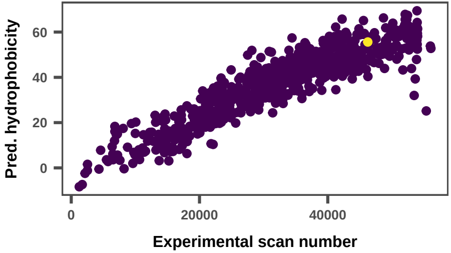

C

Distributions of residuals from best−fit line
of predicted RT vs Expt. scan number
Line: Z score of novel peptide
Z: 0.185

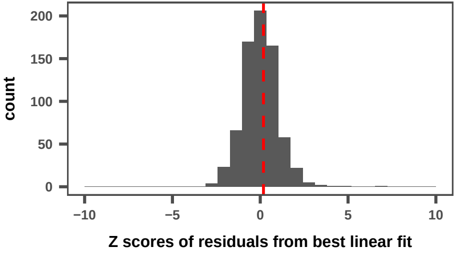

Supplement: 2 [file NIHMS1546469-supplement-2.zip › DF1/PXD010154/Heart/Heart_45_MAP4_MYHDDDLADLVFPSSATADTSIFAGQNDPLKDSYVPLELAK.pdf]
